# Light Pollution and Circadian Misalignment: A Healthy, Blue-Free, White Light-Emitting Diode to Avoid Chronodisruption

**DOI:** 10.3390/ijerph19031849

**Published:** 2022-02-07

**Authors:** Amador Menéndez-Velázquez, Dolores Morales, Ana Belén García-Delgado

**Affiliations:** Photoactive Materials Research Unit, IDONIAL Technology Center, 33417 Avilés, Spain; dolores.morales@idonial.com (D.M.); ana.delgado@idonial.com (A.B.G.-D.)

**Keywords:** light pollution, artificial light at night (ALAN), circadian rhythms, chronodisruption, white light-emitting diode (WLED), blue-free WLED, color reproduction index (CRI), luminescent organic materials, spectral converters

## Abstract

Sunlight has participated in the development of all life forms on Earth. The micro-world and the daily rhythms of plants and animals are strongly regulated by the light–dark rhythm. Human beings have followed this pattern for thousands of years. The discovery and development of artificial light sources eliminated the workings of this physiological clock. The world’s current external environment is full of light pollution. In many electrical light bulbs used today and considered “environmentally friendly,” such as LED devices, electrical energy is converted into short-wavelength illumination that we have not experienced in the past. Such illumination effectively becomes “biological light pollution” and disrupts our pineal melatonin production. The suppression of melatonin at night alters our circadian rhythms (biological rhythms with a periodicity of 24 h). This alteration is known as chronodisruption and is associated with numerous diseases. In this article, we present a blue-free WLED (white light-emitting diode) that can avoid chronodisruption and preserve circadian rhythms. This WLED also maintains the spectral quality of light measured through parameters such as CRI (color reproduction index).

## 1. Introduction

In 1609, Galileo Galilei pointed a telescope at the night sky. His inspection of the pathways of stars and planets brought new and profound knowledge of celestial movement. His discovery that the Earth is not the center of the universe propelled revolutions in science and religion [1].

Galileo’s telescopic discoveries, and the cultural commotion they caused, were remarkable. However, Galileo’s night sky was no less spectacular than his discoveries. From a contemporary perspective, it is difficult to imagine that the nocturnal world was once a central part of human existence. Starlight has aroused curiosity, inspired art, and formed the basis of countless creation stories for thousands of years. Stars have guided exploration, navigation [2], and astronomy [3], compelling some of the most significant technological leaps in human innovation.

The night sky’s transformation also serves a crucial biological purpose. Darkness helps regulate sleep patterns in humans and provides wildlife migration, feeding, and mating cues. Even trees depend on the length of the day to guide the timing of leaf molt. Cycles of light and dark have always determined the rhythms of life [4].

In large cities worldwide, millions of artificial lights have replaced natural darkness with the haze of a perpetual glow that hides all but the brightest stars. In short, light pollution is an excessive and inappropriate artificial light at night. Light pollution is increasing, covering nearly 80% of the globe [5]. Astronomers were the first to call attention to the purloined night. However, they were far from the only group affected. Our overall lives and health have also been altered by the disappearance of natural darkness [6].

Human beings are diurnal organisms whose biological clocks are governed by natural light and dark cycles [7]. We lived in harmony with these natural cycles for millennia. Throughout our evolution, our human ancestors, similar to other mammals, were also diurnal. They were generally active during daytime and rested at night under dark conditions. Bearing in mind that human evolution took place near the Equator, our ancestors followed cycles of 12 h of light and 12 h of darkness (12L–12D).

Humans have always tried to prolong daylight by whatever local means available, including burning wood, animal fat, organic and mineral oils, etc. (especially after migrating from equatorial areas to places with shorter days and longer nights). However, nighttime activities under such limited lighting sources were rather limited.

The invention of the electric light bulb changed this situation dramatically. English physicist Joseph Wilson Swan and American inventor Thomas Alva Edison publicly demonstrated this new technology almost simultaneously in 1879.

Since then, electrical light bulbs and electricity have become more reliable and affordable as energy sources for illumination. Following these technological developments, electric lights proliferated worldwide. As a result of this proliferation, humans became no longer “tied” to the traditional 12L–12D cycles. Instead, we could be active around the clock at will. Supported by artificial illumination, we can be active at night and rest during the day, contrary to our diurnal nature acquired through years of evolution.

The invention of electric light created new opportunities by allowing the extension of daylight beyond what the Sun dictated. However, exposure to artificial light at night (ALAN) inhibits the secretion of melatonin (a hormone associated with sleep) and alters our biological rhythms, causing what is known as circadian disruption or chronodisruption [8]. A growing body of scientific and clinical evidence suggests that chronodisruption is associated with numerous diseases [9], such as cancer, Alzheimer’s, and cardiovascular diseases.

In addition to its important role as a sleep regulator, melatonin has other beneficial properties for our health. It is a powerful antioxidant, an antioncogenic agent, and enhances and regulates our immune system’s proper functioning, among other properties [10]. Therefore, melatonin plays a fundamental role in the protection against diseases such as COVID, whose harmful effects on humans can be minimized with an optimal immune system [11].

Decades of research on the human eye and the discovery of a new photoreceptor in the retina, not associated with vision but with the regulation of circadian rhythms, allow us to know and understand the effects of light on our biological clocks. This photoreceptor is known as ipRGC (intrinsically photosensitive retinal ganglion cell) [12] and is sensitive to both light intensity and the spectral distribution of light. Specifically, this photoreceptor contains the pigment melanopsin, which has an absorption peak in the blue part (around 480 nm) of the visible spectrum. These ipRGC cells also receive synaptic inputs from rods and cones [13]. The results of these interactions define the spectral sensitivity of the circadian system, which reaches its maximum in the wavelength range from about 430 nm to 500 nm.

White light-emitting diodes (WLEDs) are energy efficient and more environmentally friendly than their predecessors. In any case, the white light they emit contains a large proportion of blue light in the spectral range between 430 nm and 500 nm. This blue light interacts with the ipRGC photoreceptors and inhibits the secretion of melatonin with a consequent alteration of biological rhythms [14].

We cannot regress to Edison’s incandescent light bulb, which is inefficient from an energy perspective. However, we can move toward lights that simultaneously preserve energy efficiency, spectral quality, and human health. In this article, we propose a light bulb that meets these characteristics. We developed a blue-free WLED to avoid chronodisruption while maintaining high quality light parameters, such as the color rendering index (CRI). To our knowledge, this is the blue-free WLED based on organic materials with the highest CRI. This is a proof of concept which allows us to conclude that it is possible to design truly efficient and healthy WLED lights that support the body’s biological needs (non-visual effects of light) and are good at rendering the color of objects (visual effects of light).

## 2. Materials and Methods

Most white LEDs on the market use blue LED chips manufactured from GaN-based materials and a phosphor coating placed either directly on the chips or separated from the chip (remote phosphor configuration) [15]. A phosphor is defined as a luminescent material that absorbs and reemits energy over time, which is associated with the lifetime of the excited electron. A fraction of the blue light passing through the phosphor is absorbed, experiences the Stokes shift down-shifting, and appears as white light when mixed with the remaining unabsorbed blue light. Shuji Nakamura first demonstrated this and was awarded with the Nobel prize for his blue and white LED inventions [16,17].

These WLED sources emit light with characteristic spectral distributions that depend on the nature of the excitation source (blue LED chip) and the phosphors (spectral converters) used. Most phosphors are inorganic, i.e., carbon-free crystals. Furthermore, many of them are based on rare earths subject to geopolitical tensions. Therefore, the search for alternative luminescent materials is a promising alternative. Quantum dots [18,19,20,21] and organic dyes [22] attract attention due to their high photoluminescence quantum yields and tunable light emission. They may be used as an alternative to traditional phosphors for allowing a fine adjustment of CRI and CCT.

By showing a narrow emission leading to pure colors, quantum dots have been implemented in commercial flat panel displays. Organic luminescent dyes show a broader emission. Therefore, they can be very useful for general lighting in order to cover the whole visible spectrum. Usually, the absorption spectrum of quantum dots is broader than the absorption spectrum of organic dyes. However, this is not a problem for organic dyes if their absorption spectrum covers the emission range of the LED chip acting as a source of excitation.

In summary, quantum dots and organic dyes are promising materials as color converters for lighting. However, the most efficient QDs are based on toxic Cd. Organic dyes are sustainable, environmentally friendly, and cheaper than quantum dots.

We present a study of WLEDs using organic fluorescent dyes to replace the conventional phosphor. These organic luminescent materials have already been tested as spectral converters in the energy sector (photovoltaics) [23,24,25], in the agrifood sector (greenhouses) [26], and even in the medical sector (ophthalmology) [27] to achieve a specific spectral redistribution. The lighting industry may benefit from these types of materials, which are sustainable, environmentally friendly, and free of geopolitical tensions.

We developed different blue-free WLEDs for this article. They are based on blue LED chips and luminescent organic materials in a remote phosphor configuration acting as spectral converters. These luminescent organic materials combine in the same layer or different layers in order to have greater versatility in the spectral conversion, thus achieving an emitted light with the desired properties.

### Selected Excitation Source and Materials

**Figure 1:**
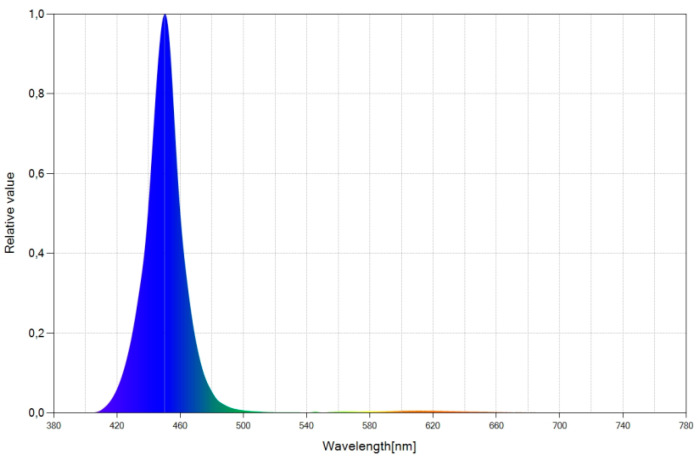
Spectral power distribution (SPD) chart of the selected blue LED.

As excitation source, we used an LED whose relative spectral power distribution (SPD) chart is shown in Figure 1. The SPD provides emitted power as a function of the wavelength. The LED emits light in the wavelength range between 410 nm and 500 nm, corresponding to the blue range of the spectrum, with an emission peak at 450 nm.

The luminescent organic molecules are incorporated into a polymer matrix with high optical transparency, such as PMMA. These composite systems (polymeric matrix and luminescent species) are deposited on glass in the form of thin films. These thin films function as luminescent filters that spectrally convert light and expand the spectrum of the emitted light with respect to the blue LED chip, in order to cover a larger fraction of the visible part of the spectrum.

**Figure 2:**
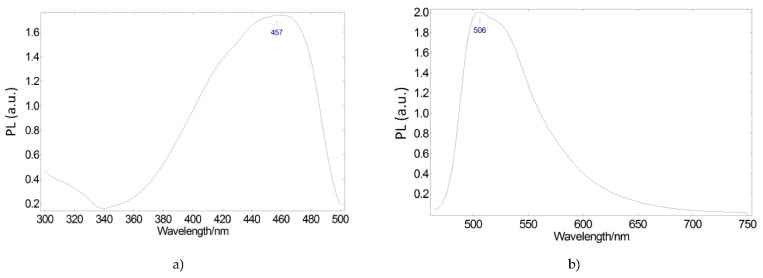
Photoluminescent excitation (**a**) and emission (**b**) spectra of Coumarin 6 green-emitting converter embedded in a PMMA matrix.

Regarding the luminescent species, we sought organic dye molecules whose excitation spectral range covers the emission spectrum of the selected blue LED. The Coumarin 6 dye meets this requirement. Figure 2 shows its excitation and emission spectra. The visible excitation spectrum (Figure 2a) extends from ultraviolet to 500 nm, with an excitation peak at 457 nm, very close to the emission peak of the blue LED. Likewise, the emission spectrum under the excitation wavelength of 457 nm presents a peak at 506 nm (Figure 2b), allowing the extension of the blue LED + phosphor system’s emission range with respect to the pure blue LED.

**Figure 3:**
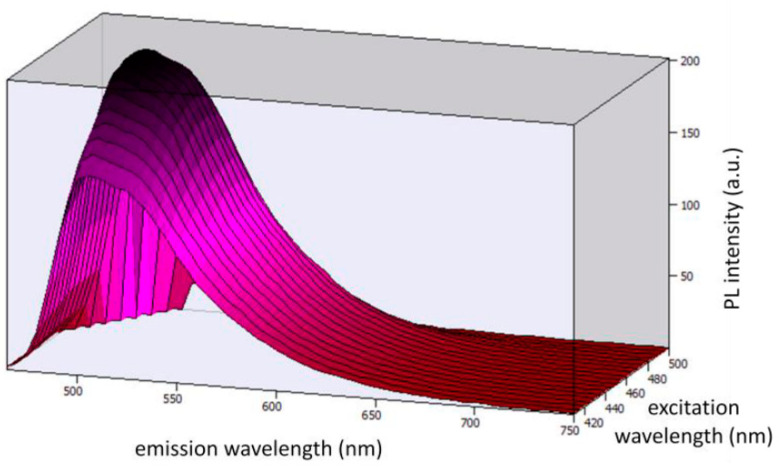
3D photoluminescent spectra of Coumarin 6 embedded in a PMMA matrix under the excitation wavelengths from 410 nm to 500 nm.

Figure 3 shows the 3D photoluminescent (PL) spectra under the excitation wavelengths from 410 nm to 500 nm (corresponding to the emission range of the blue LED chip). By observing the spectra, we can conclude that Coumarin 6 embedded in PMMA can be effectively excited by the selected blue LED chip.

**Figure 4:**
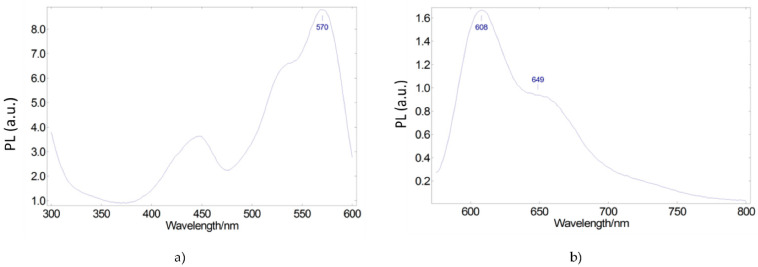
Photoluminescent excitation (**a**) and emission (**b**) spectra of Lumogen Red red-emitting converter embedded in a PMMA matrix.

We then sought a second luminescent species that allowed us to extend the spectral range of emitted light further. Lumogen Red is a molecule with a broad excitation spectrum that can partially absorb light emitted by the blue LED and light corresponding to the Coumarin 6 emission spectrum. Figure 4 shows its excitation and emission spectra. The visible excitation spectrum (Figure 4a) extends from ultraviolet to 600 nm with an excitation peak at 450 nm, which overlaps with the emission peak of the blue LED. Another excitation peak at 570 nm overlaps with the Coumarin 6 emission spectrum. Likewise, the emission spectrum under the excitation wavelength of 570 nm presents two peaks at 608 nm and 649 nm (Figure 4b), allowing the extension of the spectral range of the emitted light.

**Figure 5:**
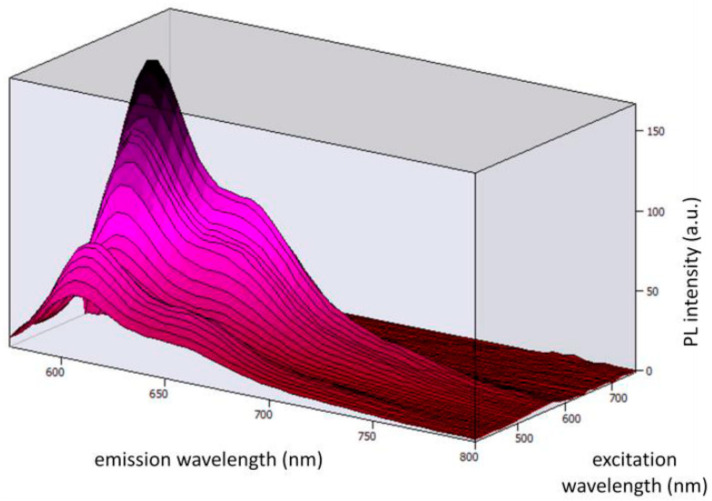
3D photoluminescent spectra Lumogen Red embedded in a PMMA matrix under the excitation wavelengths from 410 nm to 740 nm.

Figure 5 shows the 3D PL spectra under the excitation wavelengths from 410 nm to 740 nm (corresponding to the emission of the blue LED chip and the Coumarin 6 dye). By observing the spectra, we can conclude that Lumogen Red embedded in PMMA can be excited by the blue LED + Coumarin 6 system.

Another key property of a luminescent material acting as spectral converter is the quantum yield (QY), which is defined as the ratio of emitted photons to absorbed photons. These luminescent organic dyes (Coumarin 6 and Lumogen Red) possess high quantum yields both in solution and solid state. QY of Coumarin 6 and Lumogen Red embedded in PMMA is 76% and 97%, respectively.

## 3. Results and Discussion

By combining the luminescent species Coumarin 6 (C6) and Lumogen Red (LRed) in the same layer or different layers and varying their concentrations, it is possible to achieve different spectral conversions. The different concentrations are expressed as percent by weight with respect to the host material (PMMA).

We developed 19 different WLEDs from the previously described blue LED chip and 19 different spectral converters. We grouped the different spectral converters into three categories: monolayer molecular systems with one luminescent dye (Section 3.2), monolayer molecular systems with two luminescent dyes (Section 3.3), and multilayer luminescent molecular systems (Section 3.4). It is possible to compare the different WLEDs and optimize their configuration to achieve a blue-free WLED with high light quality by measuring their optical properties. In Section 3.1, we describe and justify the optical characterizations that will be carried out in the following sections with the different WLEDs we developed.

### 3.1. Optical Characterization

#### 3.1.1. Spectral Distribution Power (SPD)

The spectral power distribution (SPD) chart visually represents the light spectrum emitted by a light source. It provides emitted power as a function of the wavelength. SPD charts are a practical way to compare the quality and type of light created by different light sources. The chart shows which wavelengths of light (measured in nanometers) the illuminated regions receive. Therefore, it can be useful to visualize the fraction of blue light that different LEDs emit.

#### 3.1.2. Color Rendering Index (CRI)

The color rendering index, also known as the CRI or Ra value, is defined by the Commission Internationale de l’Éclairage (CIE, International Commission on Illumination) as a quantitative indicator of a light source’s ability to correctly reproduce the R_i_ test colors of any object in comparison to a reference light source, such as sunlight. The standard CRI points, namely R_1_−R_8_, derive from CIE 1974 test color samples. The value of CRI is calculated as CRI = ΣR_i/_8. CRI ranges from 0 (no color rendering) to 100 (perfect color rendering). The special CRI points R_9_–R_15_ are also color rendering markers represented in the CRI points diagrams.

#### 3.1.3. Correlated Color Temperature (CCT)

The emission spectrum of a blackbody radiator is a function of its temperature. An arbitrary white light source can be characterized by finding the temperature of the blackbody radiator that radiates light of a color comparable to that of the light source. This temperature is called the correlated color temperature (CCT) and is measured in Kelvin (K). For example, the color of a candle’s flame is similar to a black body heated to about 1800 K. The flame is said to have a “color temperature” of 1800 K. Similar to other incandescent bodies, the black body changes its color with increased temperature. It first acquires a red tone, then changes to orange, yellow, and finally white, bluish-white, and blue.

Although the correlation is not always perfect, the lowest color temperatures generally correspond to warm lights with low blue content, and the highest color temperatures correspond to cool lights with high blue content. Therefore, color temperature is a parameter we can also consider when designing LEDs with low blue content.

### 3.2. Monolayer Molecular Systems with One Luminescent Dye

We developed four WLEDs from the blue LED chip (See Figure 1) by integrating an organic luminescent dye into a single layer and using different concentrations of the dye for each WLED. Table 1 summarizes the characteristics of the WLEDs we developed.

**Figure 6:**
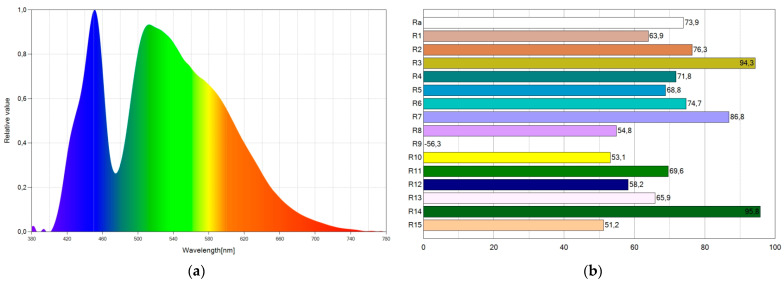
Spectral power distribution chart (**a**) and CRI points (**b**) of WLED4.

As shown in Table 1, white light with a CRI above 70 was achieved using Coumarin 6 dye with a concentration of 2.3%. However, the blue component is still significant, even in WLED4, which has the highest dye concentration and therefore the greatest capacity to absorb blue light. This result is shown in Figure 6a with the spectral distribution power of WLED1. The SPD shows two peaks in the spectrum, corresponding to the emission peak of the blue LED chip and the emission peak of the Coumarin 6 dye. Figure 6b shows the corresponding CRI points.

### 3.3. Monolayer Molecular Systems with Two Luminescent Dyes

We developed three WLEDs from the same blue LED chip (see Figure 1) and two luminescent species (Coumarin 6 and Lumogen Red) integrated into the same layer. By using two luminescent dyes, instead of just one luminescent dye, we pursued a double objective: on the one hand, to improve the CRI by expanding the emission spectrum; on the other hand, to reduce the blue peak with the contribution of two luminescent species, which present some absorption around the blue LED emission peak (450 nm). We also increased the concentration of the Coumarin 6 dye to help further reduce the blue peak of the spectrum.

Table 2 shows different formulations for these organic dyes and the CRI and CCT values of the corresponding WLEDs. WLED6 achieved a CRI higher than 75, thus improving the CRI of the WLEDs developed with a single luminescent species in the previous section.

**Figure 7:**
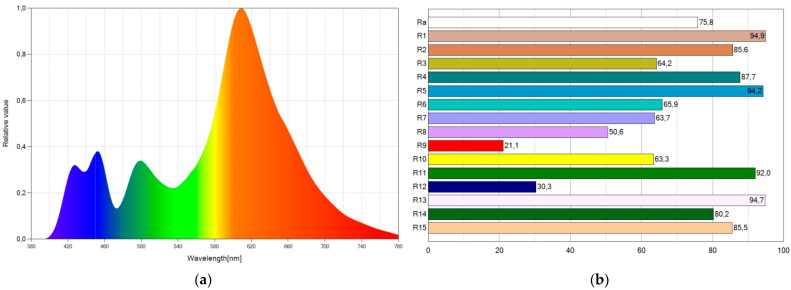
Spectral power distribution chart (**a**) and CRI points (**b**) of WLED6.

WLED6 reduces the intensity of the blue peak of the spectrum most out of all the WLEDs. Figure 7 shows the SPD chart and the CRI points of WLED6. As shown in Figure 7a, WLED6 reduces the intensity of the blue peak by more than half with respect to WLED4. It also improves its CRI by almost two units (see Figure 6b and Figure 7b).

### 3.4. Multilayer Luminescent Molecular Systems

We developed multilayer WLEDs to reduce further and practically eliminate the blue peak of the WLEDs and improve CRI values. The molecular systems of Section 3.3 (corresponding to WLED5, WLED6, and WLED7), formed by two luminescent species, combine with the molecular systems of Section 3.2 (corresponding to WLED1, WLED2, WLED3, and WLED4), formed by a luminescent species, to create 12 new WLEDs. The molecular systems from Section 3.3 integrate into layer 1 (closest to the blue LED) of the new WLEDs and the molecular systems from Section 3.2. integrate into layer 2 (the most distant from the blue LED). Table 3 shows different WLED configurations and the corresponding CRI and CCT values. CRI values improve significantly, surpassing the value of 80 in all WLEDs. It also significantly lowers the value of the color temperature, which is generally correlated with a lower proportion of blue light, as mentioned before.

**Figure 8:**
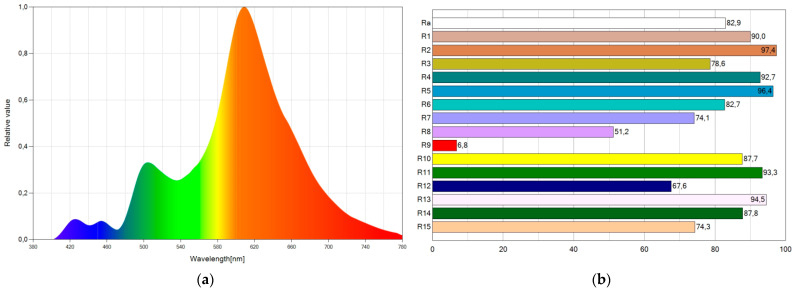
Spectral power distribution chart (**a**) and CRI points (**b**) of WLED8.

Figure 8 shows the SPD chart and CRI points of the WLED with the best CRI value (82.9), corresponding to WLED8. There are high CRI values (Figure 8b) and hardly any blue component of emitted light (Figure 8a).

**Figure 9:**
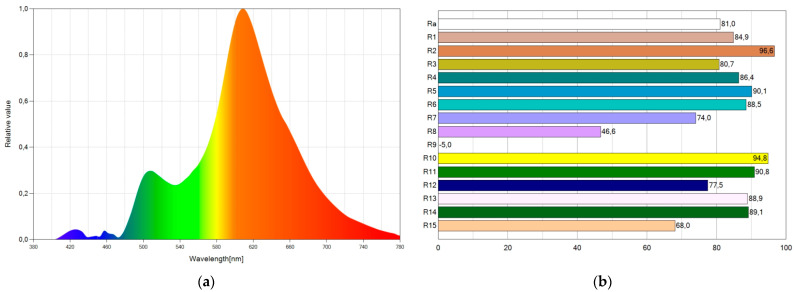
Spectral power distribution chart (**a**) and CRI points (**b**) of WLED11.

It is possible to lower the blue component and color temperature using WLED11, which marginally sacrifices the CRI a little bit. The CRI assumes a value of 81.0, and the color temperature has a value of 2080 K. Figure 9 shows the SPD chart and CRI points of WLED11. The blue emission is minimal (Figure 9a), and the CRI values are high (Figure 9b).

Therefore, two WLEDs with high CRI values and minimal blue color emissions (corresponding to a very low CCT) were achieved. WLED8 had the best CRI value (82.9) and a color temperature of 2187 K. WLED11 had the lowest CCT value (2080 K) and a CRI of 81.0.

## 4. Conclusions

In recent decades, overall living conditions and healthcare have improved considerably in many regions of the world. On the other hand, new emerging factors, such as air pollution, seriously endanger our health. In this article, we consider and draw attention to potentially harmful agents such as light. How can light become a risk factor for human beings? We are certainly not talking about natural light provided by the Sun, which living beings have become accustomed to after years of evolution. Instead, we refer to the potentially harmful effects of exposure to artificial light at night (ALAN). ALAN differs from sunlight in several important ways: the time and duration of exposure, intensity, and the spectral distribution or range of wavelengths of emitted light.

We are currently searching for lighting devices that provide lighting efficiency and are energy-saving. Simultaneously, we should consider the biological effects of these lights. The light emitted by these luminaires contains a large fraction of blue light despite being perceived as white. This blue light interferes with our circadian rhythm during the night, causing misalignment and depriving us of restful sleep. To overcome this problem, we developed an LED luminaire that emits high-quality white light with hardly any blue component. The color temperature is 2080 K, which corroborates that the emitted light is warm with very low blue radiation. This luminaire also has a high CRI (81.0), guaranteeing good visual perception. Furthermore, it is manufactured from sustainable organic materials that respect the environment. Contrary to many rare-earth-based LED luminaires, it is free of geopolitical tensions. To the best of our knowledge, this is the first luminaire to simultaneously comply with these characteristics, care for human health and the planet.

## Figures and Tables

**Table 1:** Description of the WLEDs (1, 2, 3, and 4) and their optical properties.

WLED	% Dye (Layer 1)	CRI	CCT (K)
WLED1	C6 1.5%	-	-
WLED2	C6 2.3%	70.6	7997
WLED3	C6 2.5%	73.7	8627
WLED4	C6 3%	73.9	6895

**Table 2:** Description of the WLEDs (5, 6, and 7) and their optical properties.

WLED	% Dye (Layer 1)	CRI	CCT (K)
WLED5	C6 4% LRed 0.75%	73.6	2388
WLED6	C6 5% LRed 0.75%	75.8	2292
WLED7	C6 6% LRed 0.75%	72.8	2369

**Table 3:** Description of the WLEDs (8–19) and their optical properties.

WLED	% Dye (Layer 1)	% Dye (Layer 2)	CRI	CCT (K)
WLED8	C6 4% LRed 0.75%	C6 1.5%	82.9	2187
WLED9	C6 4% LRed 0.75%	C6 2.3%	81.6	2124
WLED10	C6 4% LRed 0.75%	C6 2.5%	81.5	2113
WLED11	C6 4% LRed 0.75%	C6 3%	81.0	2080
WLED12	C6 5% LRed 0.75%	C6 1.5%	82.9	2167
WLED13	C6 5% LRed 0.75%	C6 2.3%	81.3	2110
WLED14	C6 5% LRed 0.75%	C6 2.5%	81.2	2111
WLED15	C6 5% LRed 0.75%	C6 3%	80.2	2078
WLED16	C6 6% LRed 0.75%	C6 1.5%	82.1	2174
WLED17	C6 6% LRed 0.75%	C6 2.3%	81.3	2096
WLED18	C6 6% LRed 0.75%	C6 2.5%	81.4	2107
WLED19	C6 6% LRed 0.75%	C6 3%	80.9	2083

## Data Availability

The data supporting the findings of this study are available within the article.
